# Concomitant injuries in patients with thoracic vertebral body fractures—a systematic literature review

**DOI:** 10.1007/s00402-021-03830-2

**Published:** 2021-03-01

**Authors:** Ulrich J. Spiegl, Georg Osterhoff, Philipp Bula, Frank Hartmann, Max J. Scheyerer, Klaus J. Schnake, Bernhard W. Ullrich

**Affiliations:** 1 Department of Orthopaedics, Trauma Surgery and Plastic Surgery, University of Leipzig, Liebigstr. 20, 04103 Leipzig, Germany; 2 Department of Orthopaedics and Trauma Surgery, Klinikum Gütersloh, Gütersloh, Germany; 3 Center for Trauma and Orthopedic Surgery, Gemeinschaftsklinikum Mittelrhein, Ev. Stift, Koblenz, Germany; 4 Department of Orthopedics and Trauma Surgery, University Hospital of Cologne, Cologne, Germany; 5 Center for Spinal and Scoliosis Surgery, Malteser Waldkrankenhaus, St. Marien, Erlangen, Germany; 6 Department of Trauma Surgery and Reconstructive Surgery, BG Klinikum Bergmannstrost, Halle, Germany

**Keywords:** Thoracic spine fractures, Additional thoracic injuries, Neurologic deficit, Timing of surgical stabilization

## Abstract

**Purpose:**

The aim of this study was to give a systematic overview over the rate and location of concomitant injuries, the probability of suffering from neurological deficits, and to give evidence of the timing of surgery in severely injured patients with unstable thoracic vertebral body fractures*.*

**Methods:**

This review is based on articles retrieved by a systematic search in the PubMed and Web of Science database for publications up to November 2020 dealing with unstable fractures of the mid-thoracic spine.

**Results:**

Altogether, 1109 articles were retrieved from the literature search. A total of 1095 articles were excluded. Thus, 16 remaining original articles were included in this systematic review depicting the topics timing of surgery in polytraumatized patients, outcome neurologic deficits, and impact of concomitant injuries. The overall level of evidence of the vast majority of studies is low.

**Conclusion:**

The evidence of the available literature is low. The cited studies reveal that thoracic spinal fractures are associated with a high number of neurological deficits and concomitant injuries, particularly of the thoracic cage and the lung. Thereby, diagnostic algorithm should include computer tomography of the whole thoracic cage if there is any clinical sign of concomitant injuries. Patients with incomplete neurologic deficits benefit from early surgery consisting of decompression and long-segmental stabilization.

## Introduction

The majority of articles dealing with thoracolumbar fractures are focusing on the thoracolumbar junction (TLJ) including the region between the 11th thoracic vertebral body and the 2nd lumbar vertebral body [21]. This is not surprising considering the distribution of vertebral fractures at the thoracolumbar spine. Reinhold et al. [14] included 865 patients with fractures of the thoracolumbar spine and reported 69% of the fractures at the thoracolumbar junction, whereas only 18% of the fractures were located between the 1st and the 10th thoracic vertebral body (thoracic spine). This can be explained by the protective effect of the rib cage [20]. However, the rate of severe fractures with a high grade of instability defined by type B and C fractures is 71.5% at the thoracic spine. This rate substantially exceeds the rates of highly unstable fractures at the TLJ and the lumbar spine [14]. Thus, mainly high-energy trauma seems to be responsible for the majority of thoracic vertebral fractures in adult patients with healthy bone stock. However, this increases the risk of concomitant injuries such as the thoracic cage and the spinal cord [19]. This can have consequences on the diagnostic algorithm in order to avoid missing concomitant injuries and of the timing of the operative treatment in these patients.

Therefore, the aim of this study is to give a systematic overview of the rate and location of concomitant injuries, the probability of suffering from neurological deficits, and to give evidence of the timing of surgery in severely injured patients with unstable thoracic vertebral body fractures*.*

## Methods

The literature search included unstable recent vertebral fractures (< 4 weeks) of the thoracic spine (Th 1–Th 10) of adults with adequate trauma history. Children and adolescents (age < 18) and elderly (age > 65) with likely concomitant osteopenia/osteoporosis were not within the scope of this review and need to be analyzed separately. Furthermore, patients with fractures after non-adequate trauma (trivial falls from tripping) were not included in this review.

A systematic search of the literature was performed by two of the authors (UJS, BWU), including all articles until 05/11/2020. In each case, the two databases PubMed and Web of Science Core Collection were considered and searched. Excluded were articles dealing with osteoporotic or pathologic vertebral body fractures, cervical and/or lumbar vertebral body fractures, and exclusively non-operative therapy strategies. Additionally, case reports, reviews, and animals studies were excluded. Since data collection had already been completed at the time of PROSPERO registration, this review could not be registered with PROSPERO. Using the PICO scheme [3] the following review questions were defined:

- What concomitant injuries can be expected in patients with thoracic spine injuries?
- How many patients suffer from neurologic deficits caused by thoracic vertebral body fractures and what is the expected course of it?
- When to operate in polytraumatized patients?

The following search terms were used: “thoracic vertebral body fractures” OR “thoracic vertebral spine fractures” NOT “Osteoporosis” NOT “case report” NOT “tumor” NOT “lumbar spine”.

Subsequently, all relevant original articles were analyzed based on their levels of evidence and their appropriate conclusions. Here, the following topic areas were defined:

- Neurologic deficits
- Impact of concomitant injuries
- Timing of surgery in polytraumatized patients

## Results

**Fig. 1:**
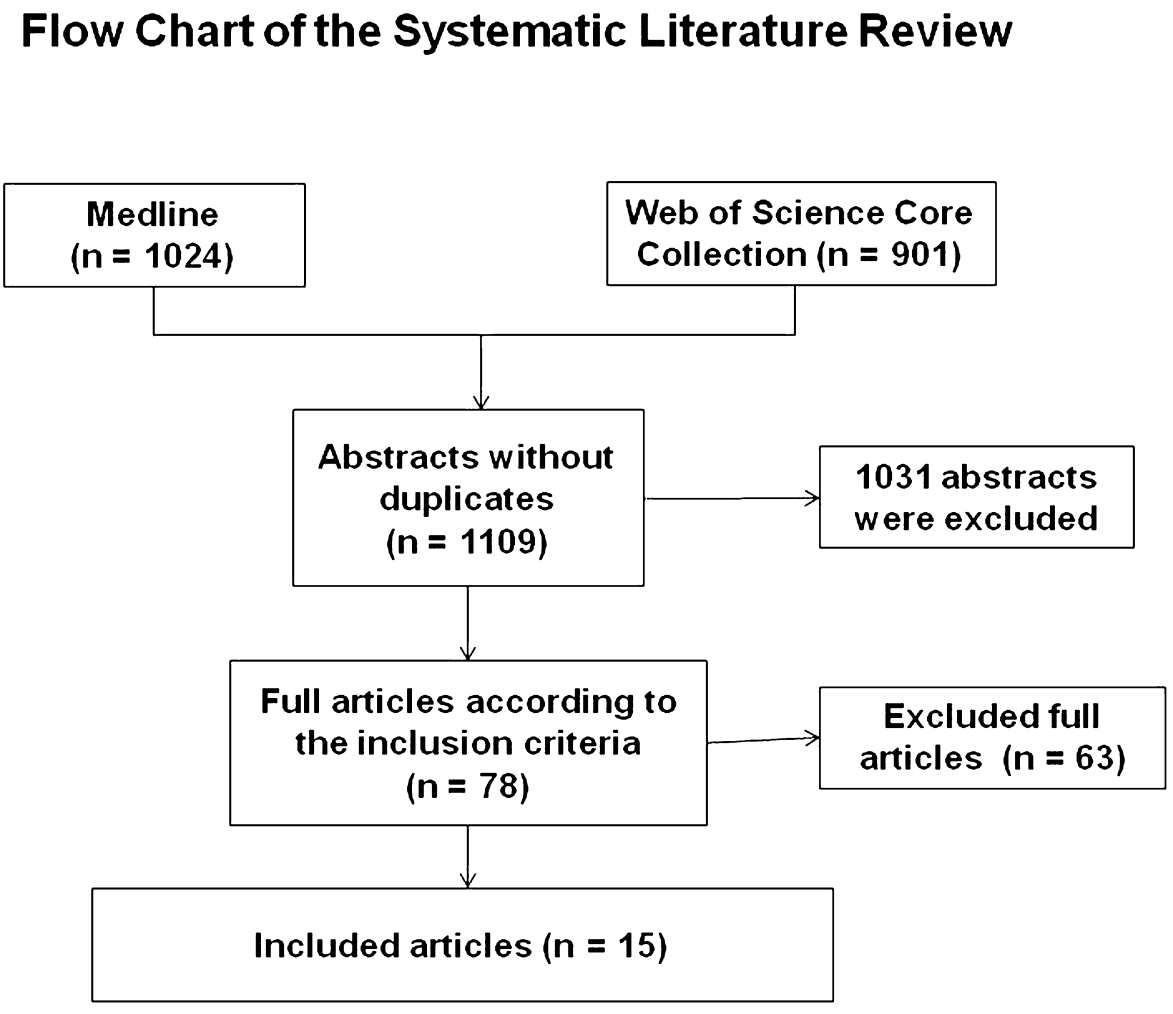
Flowchart of the systematic literature review

Altogether, 1109 abstracts were retrieved from the literature search (Fig. 1). Of these, articles were excluded based on abstract or title. Most of the excluded studies were overlaps between both databases, animals studies, no original articles or were articles investigated other pathologies or included cervical or lumbar factures, or exclusively evaluated non-operative treatment or anterior approaches. Altogether, 78 articles were analyzed completely. Of these articles 26 were additionally excluded, not focusing specifically on the thoracic spine, including geriatric patients or insufficiently describing the method of posterior stabilization. A total of 36 articles analyzed the technique of pedicle screw placement, the biomechanics of posterior stabilization of midthoracic fractures as well as the outcome of patients suffering from midthoracic fractures, and were excluded. Altogether, *1096* articles were excluded (Fig. 1). All *15* remaining original articles, which covered the period from 1971 to 2020 are summarized in Tables 1, 2, 3 and 4. Levels of evidence were defined as described by Bassler and Antes [1].

**Table 1:** Studies dealing with additional thoracic injuries

References	Purpose	Study design	N	FU (months)	Main message	Ev-L
Huang et al. [5]	Analysis of middle and upper thoracic fractures associated with sternal fractures	Retrospective case series	26	8–99	Middle and upper thoracic spine fractures with associated sternal fractures are caused by high-energy injuries and have a high rate of unstable fracture pattern	IV
Krinner et al. [8]	Anterior sternal plating in patients with unstable vertebral spine fractures and associated sterna fractures	Retrospective case series	11	24	Sternal plating with low profile plates was associated without any complications with complete consolidation after 3 months in all cases	IV
Lemburg et al. [9]	Impact of CT findings of the chest wall, mediastinum, lungs, and pleural space on the mortality	Retrospective case series	33	Hospital stay	Survivers had a significant lower lung contusion scores Chest wall bruises, rib fractures, mediastinal hematoma, and bilateral pneumothoraces was assiocated with higher mortality rates	IV
Scheyerer et al. [16]	Impact of sternal fracture location on concomitant injuries	Retrospective case series	58	None	Fractures of the manubrium sterni had the highest rate of concomitant injuries Sternal fractures are associated with serios injuries of the chest wall, thoracic spine factures, and brain injuries	IV
Wang et al. [22]	Clinical characteristics of patients with vertebral spine fractures and concomitant fractures of the ribs	Retrospective cohort study	226	Hospital stay	Patients with thoracic vertebral body fractures had a higher frequency of multiple rib fractures Increased number of rib fractures was associated with prolonged intensive care stay and increased frequency of pulmonary complications	IV
Morgenstern et al. [12]	Correlation between sternal fractures and unstable thoracic spine injuries	Retrospective cohort study	64	None	A thoracic cage injury was significantly more frequently associated with a highly unstable thoracic spine injury	IV

**Table 2:** The percentage and number of additional concomitant injuries are summed

Article	Rib fract % (*n*)	Sternal fract % (*n*)	Lung cont % (*n*)	Pneumo-/Hemato-thorax % (*n*)	Aortic lesion % (*n*)	Cranio-cerebral injuries % (*n*)	Abd. visceral injuries % (*n*)
Wang et al. [22]	22 (226)	2 (5)	28 (64)	11 (24)	0	11 (24)	4 (8)
Morgenstern et al. [12]	64 (41)	10 (206)	64 (41)	42 (54)	2 (1)		
Lemburg et al. [9]	30 (10)	9 (3)	64 (21)	24 (8)	0		
Huang et al. [5]	100 (26)		31 (8)	46 (12)			8 (2)

*Fract.* Fracture, *cont.* contusion, *abd.* abdominal

**Table 3:** Articles dealing neurologic deficits

References	Purpose	Study design	*n*	FU (months)	Main Message	Ev-L
Krengel et al. [7]	Prognosis of patients with incomplete neurologic deficit and early stabilization with decompression	Retrospective case series	14	20	Average neurologic improvement was 2.2 Frankel grades 64% had no deficits and final follow-up	IV
Place et al. [13]	Comparison of operative with non-operative treatment of patients with complete paraplegia	Retrospective case series	113	> 60	Longer inpatient stay in rehabilitation of patients treated non-operatively	IV
Sapkas et al. [15]	Outcome after stabilization and decompression within 4 days after trauma	Retrospective case series	29	12–180	All patients with Frankel A status preoperatively remained their status All others improved by average of 1.5 Frankel scores	IV
Sobottke et al. [18]	Outcome after long-segmental posterior stabilization with decompression	Retrospective case series	60	2–19	Reduction loss was 4.7° after posterior only long-segmental stabilization No anterior might be necessary	IV

**Table 4:** Articles dealing with the timing of stabilization of the thoracic spine in polytraumatized patients

References	Purpose	Study design	*n*	FU (months)	Main message	Ev-L
Schinkel et al. [17]	Impact of early spine stabilization within 72 h in mortality and hospital stay in patients with severe thoracic injuries	Retrospective case series	298	3–12	Early stabilization was associated with a shorter intensive care unit stay, shorter mechanical ventilation, and shorter hospital stay Reduced expected mortality after early stabilization	IV
Lubelski et al. [10]	Impact of very early spine stabilization within 36 h on the outcome with additional severe thoracic injuries	Prospective case series	340	Hospital stay	Significant lower complication rate after early treatment	III
Frangen et al. [4]	Impact of early spine (72 h) stabilization in polytraumatized patients with severe thoracic injuries	Retrospective case series	160	Hospital stay	No effect for patients with minor thoracic injuries Beneficial effect for patients with severe thoracic injuries with fewer pulmonary complications, shorter ventilator support, shorter hospital stay	IV
Konieczny et al. [6]	Impact of early thoracic spine stabilization within 3 days after trauma	Prospective case series	38	Hospital stay	Early operated patients in severe thoracic trauma and low initial Hb levels (< 10 g/dL) and/or a thoracic drain may pose a risk for poor outcome	III

### Impact of additional thoracic injuries

Six studies analyzed the percentage of additional thoracic injuries in combination with thoracic spine fractures (Table 1). In this context, an important but frequently overlooked combination is that of thoracic spinal injury and rib fractures. Those are commonly associated with pulmonary contusions, pneumothorax, and lung injury which may significantly contribute to the pulmonary morbidity and adverse impact on the patients` outcome [9]. Wang et al. [22] reported an incidence of 7.2% of rib fractures associated with traumatic spinal fractures. With a focus on thoracic spinal involvement the incidence increased up to 13.1% and in cases with unstable thoracic vertebral fractures up to 30% [9, 22]. Besides, the combination of thoracic spinal injury and rib fracture is a significant predictor for other more serious injuries and adverse events, like pulmonary complications, neurological deficits, other bone fractures, craniocerebral injuries, and death [9]. However, even more important is the combination of vertebral fractures and concomitant fractures of the sternum with an incidence of about 30% after thoracic spine fractures [8]. In most cases mechanism of injury is a high-energy deceleration trauma caused mainly by road traffic accidents and falls [5, 16]. Morgenstern et al. [12] demonstrated that concomitant fractures of the sternum are an indicator for unstable injuries of the spine, particularly when the sternum fracture is located in the same segment of the vertebral spine defect. Thus, rotational instability has to be expected. In contrast, displacement of the sternal fracture seems to have no influence on the severity of the thoracic spinal injury.

Additionally, in combination with unstable vertebral thoracic fractures, lung contusions are common and could be observed in 30–64% of cases followed by other lung pathologies such as pneumo- or hematopneumothorax (24–46%), pleural effusions (39%), and lacerations of the lung (6%) [5, 9]. Lesions of the lung go along with high mortality rates also due to secondary damage by posttraumatic inflammation. Besides, thoracic concomitant injuries associated with thoracic vertebral fractures are dissections or ruptures of the aorta, of the supra-aortal vessels, and of the vena cava superior [9].

### Concomitant non-thoracic injuries

The relative numbers of associated concomitant pathologies are shown in Table 2. In addition to the concomitant thoracic injuries, several further non-thoracic pathologies were frequently seen in patients with thoracic vertebral body fractures. Thereby, injuries of the extremities were particularly often observed (19% of the patients) [22]. Next, the rate of craniocerebral injuries rate of patients with thoracic vertebral fractures was 10.6% [22]. Additionally, mediastinal and abdominal visceral injuries were frequently seen (Table 2).

### Neurologic deficits

Four studies included specifically patients with complete and incomplete neurologic deficits after suffering thoracic fractures (Table 3). Generally, unstable thoracic fractures carry a high risk of neurological deficit especially in type B and C injuries according to AO Spine classification. Although not included in this review due to the unselected inclusion of all thoracolumbar fractures, a German multicenter study is worth mentioning based on the high number of patients included (637 patients). A total of 117 (18.4%) patients had thoracic injuries with 37 (32.5%) patients suffering from a neurological deficit. On admission, 22 (18.8%) patients were Frankel A, 4 (3.4%) Frankel B, 4 (3.4%) Frankel C, and 7 (5.9%) Frankel D, respectively [14].

The other studies reported in their selected patient populations complete neurological deficits (Frankel A) between 76 and 83% [15, 18]. While patients with incomplete neurological deficits often recover over time, patients with complete deficits in the vast majority of cases remain paraplegic [6, 15]. However, patients with complete neurology benefit from surgery by shorter inpatient stay in rehabilitation [13].

Early surgical intervention within 24 h including reduction, stabilization and decompression has been described in several studies [7, 15]. The reported average neurological improvement in incomplete patients was either 1.5 or 2.2 Frankel grades on average per patient. However, patients with Frankel A either remained paraplegic or had little chance of improvement [15].

### Timing of surgical stabilization in severely patients

Four studies analyzed the effect of early versus later surgery on the outcome in severely injured patients with thoracic vertebral body fractures (Table 4). Thereby, Schinkel et al. [17] analyzed the German National Trauma Database (*n* = 8057) and compared clinical parameters and outcomes of patients with severe thoracic spine injuries (Abbreviated Injury Scale > 2; *n* = 298) who underwent spine stabilization within 72 h after trauma or later. Ninety-five percent of all patients had additional severe thoracic injuries such as lung contusion. In spite of comparable demographic data, patients who underwent early surgical stabilization had a significant shorter intensive care unit stay, shorter mechanical ventilation, and shorter hospital stay. Expected mortality calculated by the “Trauma and Injury Severity Score” was significantly reduced in the group of patients who underwent surgical stabilization within 72 h but not in patients who underwent surgical stabilization later than 72 h. Similarly, Lubelski et al. [10] reported of fewer complications and better outcome after early stabilization within less than 36 h in severely injured patients with upper thoracic spine injuries.

In contrast, Frangen et al. [4] could not find a general influence of timing on clinical outcomes in their patient population. Only patients with high injury severity of the thoracic spine (SG 3–5) showed a significant benefit of an early operation within 72 h after trauma. Konieczny et al. [6] found in their prospective study of multiple injured patients with unstable fractures of thoracic spine particularly high mortality rates in patients treated surgically within 72 h after trauma with low level of hemoglobin (Hb) of less than 10 mg/dL (76%) and in patients who received a thoracic drain (75%). Based on these results, the authors concluded that although some reports indicate advantages for early surgery for thoracic spine trauma in polytraumatized patients, careful patient selection should be used and those patients with concomitant severe thoracic trauma and low initial Hb levels may pose a risk for poor clinical outcomes.

## Discussion

The majority of articles that were selected had a low level of evidence (level IV). Therefore, a narrative presentation of the results was chosen.

Based on this, the most important findings of this study are the high association of concomitant neurologic deficits and concomitant pathologies to thoracic vertebral body fractures. Both have a tremendous impact on the patient and the further treatment. Particularly, neurologic deficits are a major impairment for the patients. The rate of complete paraplegias type Frankel A was very high ranging between 19 and 83% of the patients with low chances of improvement in the further course. In contrast, those patients with incomplete neurologic deficits had good chances to benefit from early surgical intervention. Early surgery consisted of decompression and long-segmental posterior stabilization during the 24 h after accident. The average improvement of 1.5–2.2 grades, that has been reported [7, 15], can make a huge difference for the patient implying that the patient is able to walk with or without medical aids, or being dependent on a wheelchair.

Concomitant injuries to the thoracic rib cage were reported in 7–30% of the patients with thoracic vertebral fractures. Under consideration that an intact rib cage provides 40% of the stability of the thoracic spine in flexion extension, 35% in lateral bending and 31% in axial rotation this can have tremendous impact of the thoracic spine stability [23]. Sternal fractures seem to have the highest impact on stability. Watkin et al. [23] found in a biomechanical study that a sternal fracture decreases the stability of the thoracic spine significantly by more than 42% in flexion extension. This was observed clinically, leading to the postulation that the sternal-rib complex is the fourth column of the spine [2]. Several authors recommend long-segmental posterior stabilization in thoracic vertebral fractures with concomitant thoracic cage fractures [19]. Additionally, operative stabilization of concomitant sternal fractures has been discussed. The authors recommend long-segmental posterior stabilization in patients with unstable thoracic vertebral body fractures and concomitant sternal fractures. Additionally, additional plate osteosynthesis of the sternum has to be discussed critically in patients with communicated fractures of the vertebral body and high load share scores (type McCormack [11] ≥ 6) in order to minimize the chances of developing kyphotic malalignment.

Generally, due to the high rate of concomitant injuries in case of thoracic spinal fractures further diagnostic workup is imperative to optimize clinical outcome and to avoid overseeing serious and possible life-threatening lesions [5, 12, 22]. The majority of studies dealing with concomitant injuries have focused on thoracic concomitant injuries [5, 12, 22]. However, Wang et al. [22] observed a high rate of long bone fractures and craniocerebral injuries in these patients. This goes along with the high rate of high-energy accidents causing unstable thoracic spine fractures [14].

Unfortunately, the evidence level of the clinical follow-up studies is low. The timing of surgery, particularly in polytraumatized patients remains unclear [10, 17]. The majority of patients might benefit from early stabilizations within 36–72 h, whereas those patients with low hemoglobin levels as well as serious associated thoracic pathologies might benefit from surgeries at a later time point [6].

This study has several limitations. First of all articles might have been missed by the used search items and selectively including articles dealing with the thoracic spine only. Besides, the level of evidence in the majority of studies is low, leading to a limited conclusions that can be drawn out of it. Last but not least, the high number of studies with low evidence level was the reason to present the results in a narrative manner without any statistical evaluation of the strength of evidence and the precision of outcome parameters.

## Conclusions

The evidence of the available literature is low. The cited studies reveal that thoracic spinal fractures are associated with a high number of neurological deficits and concomitant thoracic injuries. A diagnostic algorithm should include a computed tomography scan of the whole thoracic cage if there is any clinical sign of concomitant injuries. Patients with incomplete neurologic deficit benefit from early surgical decompression and long-segmental stabilization.
